# Dietary partitioning of Australia's two marsupial hypercarnivores, the Tasmanian devil and the spotted-tailed quoll, across their shared distributional range

**DOI:** 10.1371/journal.pone.0188529

**Published:** 2017-11-27

**Authors:** Georgina E. Andersen, Christopher N. Johnson, Leon A. Barmuta, Menna E. Jones

**Affiliations:** 1 School of Biological Sciences, University of Tasmania, Hobart, Tasmania, Australia; 2 Australian Research Council Centre for Australian Biodiversity and Heritage, University of Tasmania, Hobart, Tasmania, Australia; University of Sydney, AUSTRALIA

## Abstract

Australia’s native marsupial fauna has just two primarily flesh-eating ‘hypercarnivores’, the Tasmanian devil (*Sarcophilus harrisii*) and the spotted-tailed quoll (*Dasyurus maculatus*) which coexist only on the island of Tasmania. Devil populations are currently declining due to a fatal transmissible cancer. Our aim was to analyse the diet of both species across their range in Tasmania, as a basis for understanding how devil decline might affect the abundance and distribution of quolls through release from competition. We used faecal analysis to describe diets of one or both species at 13 sites across Tasmania. We compared diet composition and breadth between the two species, and tested for geographic patterns in diets related to rainfall and devil population decline. Dietary items were classified into 6 broad categories: large mammals (≥ 7.0kg), medium-sized mammals (0.5–6.9kg), small mammals (< 0.5kg), birds, reptiles and invertebrates. Diet overlap based on prey-size category was high. Quoll diets were broader than devils at all but one site. Devils consumed more large and medium-sized mammals and quolls more small mammals, reptiles and invertebrates. Medium-sized mammals (mainly Tasmanian pademelon *Thylogale billardierii*), followed by large mammals (mainly Bennett’s wallaby *Macropus rufogriseus*) and birds, were the most important prey groups for both species. Diet composition varied across sites, suggesting that both species are flexible and opportunistic foragers, but was not related to rainfall for devils. Quolls included more large mammals but fewer small mammals and invertebrates in their diet in the eastern drier parts of Tasmania where devils have declined. This suggests that a competitive release of quolls may have occurred and the substantial decline of devils has provided more food in the large-mammal category for quolls, perhaps as increased scavenging opportunities. The high diet overlap suggests that if resources become limited in areas of high devil density, interspecific competition could occur.

## Introduction

Co-existence of mammalian carnivores involves complex interspecific interactions and trophic dynamics [1, 2]. Interspecific competition occurs in two ways: exploitation competition occurs when a resource unit is consumed by one species so it cannot be consumed by another; interference competition involves direct aggressive encounters (e.g. fighting) or the threat of aggression, thereby excluding a competitor from a resource [3]. Ecologically and morphologically similar species are most likely to compete. Competition could lead to exclusion of one species by another [4], but similar species can coexist in stable environments through resource partitioning [5–8]. Carnivores might partition resources by consuming prey of different sizes [9, 10], by using different habitats, or by being active at different times of the diel cycle [11]; the latter will only result in partitioning of food resource if different species of prey are active at different times of day.

Knowledge of diet is fundamental to understand the interactions among carnivore species, and their impacts on prey species [12]. Carnivore diets are influenced by the diversity, abundance and availability of prey resources, which may vary in space or time, as well as by competitive interactions with sympatric carnivore species. High dietary overlap between sympatric carnivores may indicate resource competition [10, 13–15]. This can lead to aggressive encounters and intraguild predation because carnivores searching for the same prey item are more likely to encounter one another [16, 17]. Body size influences the outcome of these interactions: typically the larger carnivore dominates and excludes the smaller carnivore [16]. Extensive dietary overlap does not necessarily result in interspecific competition, however, which is more likely to occur when a shared resource is in limited supply [18], such as during drought [19]. Determining the degree of dietary overlap is a useful first step in investigating whether resource competition might exist between sympatric carnivores.

On the island of Tasmania, Australia, the carnivore community, consists of a size-structured guild of native predators and an introduced predator. The Tasmanian devil (*Sarcophilus harrisii*) is Tasmania’s largest mammalian predator, following the extinction of Tasmania’s top predator, the thylacine (*Thylacinus cynocephalus*), in the 1930s [20]. The devil (5–14kg; [21]) co-exists with the smaller native spotted-tailed quoll (*Dasyurus maculatus*) (0.9–5kg; [22]) and the introduced feral cat (*Felis catus*), which has been part of the Tasmanian carnivore guild since the 1800s [23]. The devil is a pounce-pursuit predator [24] that is capable of short fast pursuits and hunts with a moving search [25]. It is the only Australian mammal and the only marsupial with morphological specialisations for bone-eating, and is an effective scavenger as well as predator [26]. It has a diverse diet but predominately consume larger-bodied mammals [27, 28]. The spotted-tailed quoll is an ambush predator [24] that consumes small to medium-sized mammals, among other prey [27, 29–31]. It has morphological adaptations for climbing and is the most adept arboreal carnivore in the Australian fauna [26]. The devil has undergone a severe and rapid population decline since the emergence of a novel transmissible cancer (devil facial tumour disease; DFTD), first detected in 1996 [32]. Trophic cascades following the decline of Tasmania’s largest mammalian predator could lead to ecosystem-wide changes [33–35], possibly including mesopredator release of spotted-tailed quolls [36].

We present the first comprehensive study of diet composition and overlap of Tasmanian devils and spotted-tailed quolls across their distributional range using faecal analysis. We discuss the extent to which there is potential for competition between the two species and how this might translate to a change in quoll abundance following devil decline. Rainfall is a strong bottom-up factor influencing the abundance of many prey species [33] so we expect that the east to west positive gradient in rainfall could influence the diets of, and dietary overlap between, devils and quolls. The progressive spread and severe population decline of Tasmanian devils from DFTD could affect the diets of both species, if diet composition is density-dependent in the Tasmanian devil and if the availability of prey or carcasses of prey animals for spotted-tailed quolls is affected by the density of devils. We aim to address the following specific questions: (1) what is the relative importance of the prey species consumed by devils and quolls across their sympatric range? (2) what is the diet breadth and diet overlap of these two carnivores and how can it help to understand resource partitioning patterns? (3) is there partitioning in prey-size and/or vertical niche that could minimise competition? and (4) has the population decline of Tasmanian devils from DFTD affected the diet of quolls?

## Materials and methods

### Ethics statement

This study was carried out in accordance with the University of Tasmania Animal Ethics Committee Permit #A0012361, A0016155, A0011696, A0013326 and A0015835 with permission from the Tasmanian Department of Primary Industries, Parks, Water and Environment (DPIPWE) under scientific permits TFA 12166 and TFA 13969.

### Study sites

**Fig 1:**
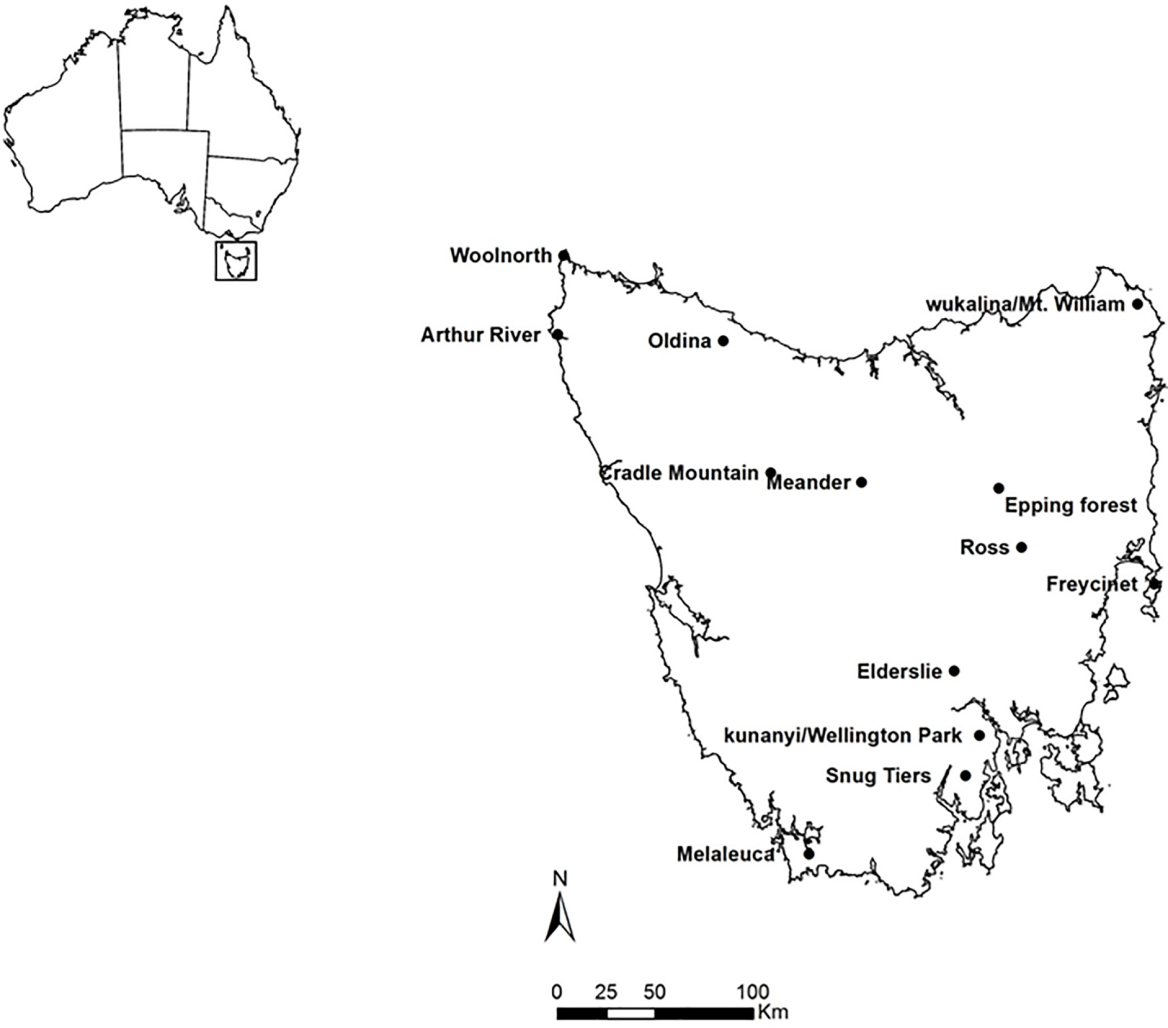
Map of study sites in Tasmania, Australia, where scats were collected.

Scats were collected from 13 sites across Tasmania (Fig 1) between 1990 and 2015, sampling the full range of environments in which devils and spotted-tailed quolls occur. Feral cats were present at all sites, but we were not able to collect enough feral cat scats to include in analyses. Tasmania has a cool temperate climate with a rainfall gradient increasing from the east to the west and south and at higher elevation, and declining temperature from low- to high-elevation regions. Study sites covered the full extent of the rainfall gradient, ranging from 423mm at Ross in the east to 2143mm at Melaleuca in the southwest (Table 1). Dominant vegetation types at each site were obtained from Reid et al. [37] and varied across sites (Table 1). At the time of scat collection, DFTD was present for a minimum of seven years and thus the devil populations would have experienced substantial decline [38], at Elderslie, Freycinet, wukalina/Mount William, kunanyi/Wellington Park, Epping Forest and Ross, and was absent at Arthur River, Woolnorth, Oldina, Snug Tiers, Meander, Melaleuca and Cradle Mountain (Table 1).

**Table 1:** Details of the site, year(s) of scat collection, location, dominant vegetation types, mean rainfall (mm) over the five years preceding the period of scat collection and year of devil facial tumour disease outbreak (DFTD) for each of the thirteen sites, where scats were collected. n = number of devil (TD) and quoll (STQ) scats collected.

Site	Year(s) of collection	n	Coordinates	Dominant vegetation	Mean rainfall (mm)	DFTD arrival
Arthur River	2012; 2013	125 (TD) 36 (STQ)	41°05´S, 144°66´E	Dry coastal vegetation; moorland and scrubland; wet eucalypt forest	1071; 1118	DFTD free
Woolnorth	2012	36 (TD)	40°69´S, 144°72´E	Wet eucalypt forest; cleared land; moorland and scrubland; farmland	771	DFTD free
Oldina	2012	28 (TD)	41°08´S, 145°67´E	Wet eucalypt forest; farmland	1318	2014
wukalina/Mount William	2012	7 (TD)	40°94´S, 148°25´E	Dry coastal vegetation; dry sclerophyll forest; woodland and native grassland	925	1995
Freycinet	2012; 2014	30 (TD) 11 (STQ)	42°20´S, 148°31´E	Dry coastal vegetation dry sclerophyll forest; woodland and native grassland	534; 439	2000
Elderslie	2012	16 (TD)	42°60´S, 147°07´E	Dry sclerophyll forest; woodland and native grassland; cleared land	961	2005
Snug Tiers	2012	27 (TD)	43°07´S, 147°26´E	Wet eucalypt forest; cleared land; dry sclerophyll forest; woodland and native grassland; farmland	1142	2014
Meander	2001	29 (TD) 19 (STQ)	41°72´S, 146°61´E	Wet eucalypt forest; cleared land; dry sclerophyll forest; woodland and native grassland; farmland	961	2003–2004
kunanyi/Wellington Park	2013; 2015	13 (TD)	42°88´S, 147°12´E	Wet eucalypt forest; dry sclerophyll forest	933; 1266	2003
Epping forest	2011	17 (STQ)	41°76´S, 147°35´E	Native grassland; dry sclerophyll forest	499	2001–2002
Ross	2011	8 (STQ)	42°03´S, 147°49´E	Native grassland; dry sclerophyll forest; farmland	423	2001–2002
Melaleuca	2014	10 (STQ)	43°42´S, 146°16´E	Wet eucalypt forest; moorland and scrubland	2143	DFTD free
Cradle Mountain	1990–1993	349 (TD) 76 (STQ)	41°68´S, 145°95´E	Wet eucalypt forest; moorlands; native grassland	2623; 2766; 2766; 2756	2004

### Scat collection and prey identification

The majority of devil and quoll scats were obtained from animals that were trapped overnight in PVC pipe traps (diameter 315mm x length 875mm) and released the next morning. Scats were stored frozen at -20°C and the species location, date, sex and individual identity (microchip number) of the animal were recorded. It is unlikely that bait consumption affected our diet analyses as traps were baited with butchered meat which leaves no residue in the scats. Scats were also opportunistically collected at some sites (kunanyi/Wellington Park, Melaleuca, Epping Forest and Ross), where we wanted representation but there were no trapping programs. These scats were identified based on size, shape, colour, odour and the presence, size and state of digestion of bone fragments. Devil scats are quite distinctive from those of quolls as only devils consume and digest large amounts of bone, which gives a grey tinge to the colour of the scats, which frequently contain sizeable shards of animal bone. We included in the analyses diet data recorded from scats collected from individually-marked trapped animals at Cradle Mountain between 1990–1993 (Jones and Barmuta [27]).

Scats were immersed in hot water for 24 hours and then washed through a 1mm sieve. Fur, feather, bone and invertebrate remains were air-dried in an oven at 60°C for 24 hours. Mammalian prey species were identified from hair, using a combination of the cross-sectional size, shape and pattern of the medulla and cortex observed at 100 and 400 times magnification under a transmission microscope; the scale patterns on the surface of the hair; and the colour, length and appearance of the hair. Identification was carried out to the lowest possible taxonomic level by comparison with known reference material and identification guides and keys [39–41]. Diet items were then classified into 6 broad categories: large mammals (≥ 7.0 kg), medium mammals (0.5–6.9 kg), small mammals (< 0.5 kg), birds, reptiles and invertebrates. The size classes for mammals are similar to those used in other Australian dietary studies [9, 30, 42]. Mammalian prey were placed in size categories based on the maximum body mass listed by Menkhorst and Knight [43]. While the majority of species can be accurately identified from hair samples, this is not always the case [44]. The two species of antechinuses that might have been represented at our study sites (dusky *Antechinus swainsonii* and swamp *A*. *minimus*) cannot be distinguished on the basis of hair samples so were grouped under their genus name. Birds, reptiles and invertebrates were not classified to species and were treated as single prey classes. We assumed that the presence of devil hair in devil samples, and quoll hair in quoll samples, was due to grooming and these were not included as prey items in analyses. The presence of quoll hair in devil samples and devil hair in quoll samples were not included in analyses as prey but were noted as evidence of intraguild consumption, probably scavenged but potentially due to predation. Remains of vegetation were also not included in analyses, as they were considered to function in digestion rather than being consumed for nutritional value [45].

### Diet composition

To ensure that we obtained a sufficient number of scats to describe the diets of each species, we calculated dietary diversity (H) using the Brillouin index [46] based on the 6 broad dietary categories described above, using the formula:

H=(ln(N!)–Σln(ni!))/N

where H is prey diversity, N is the total number of scats analysed at the site and n_*i*_ is the number of individual scat items in the *i*th category. We randomized the order of samples and plotted cumulative dietary diversity against sample size. Sample size was deemed to be sufficient if the curve reached an asymptote.

For individual prey items and prey categories, we calculated frequency of occurrence (the percentage of scats in which a certain food item was found, including as trace items) and percentage volume (the volume of a certain type of food in the scats expressed as a percentage of the total volume of all prey items in the scats). Percentage volume of each prey item in scats was estimated visually (V, estimated volume of each prey item/total estimated volume x 100) [47]. Frequency of occurrence may overestimate the dietary contribution of small mammalian prey, whereas the percentage volume may underestimate consumption of items that are easily digested. It is therefore recommended to use both metrics [12, 30].

We examined differences in the frequency of occurrence of the six prey categories between the two carnivores by pairwise comparison using chi-square contingency tests. We also pooled the frequency of occurrence of arboreal mammalian prey (brushtail, ringtail, pygmy possums and sugar gliders) *versus* ground-dwelling prey and compared the difference using a chi-square test.

### Trophic niche breadth and diet overlap

We estimated dietary niche breadth for each species across Tasmania and at each site, and diet overlap between devils and quolls, based on the use of the six dietary categories (large mammals, medium mammals, small mammals, birds, invertebrates and reptiles). At sites where information on the sex of animals was known, we estimated dietary niche breadth and overlap for each sex and species combination. Dietary niche breadth (B_A_) was calculated using Levins [48] index:

$$B_{A}=\frac{(1/\sum p_{i}^{2})-1}{n-1}$$

where p_i_ = proportion of occurrence of each prey category in the diet and n = number of possible prey categories. This measure of niche breadth ranges from 0 (narrow niche) to 1 (broad niche). Dietary overlap was calculated using Pianka’s index [7]:

$$O_{jk}=\frac{\sum p_{ij}\;p_{ik}}{\sqrt{\sum p_{ij}^{2}\sum p_{ik}^{2}}}$$

where *O* is the index of overlap, *j* and *k* are the species being compared and *p*_*i*_ is the frequency of occurrence of each dietary item. This index ranges from 0 (no overlap) to 1 (complete overlap).

### Effect of rainfall on diet composition

The effects of disease-caused population decline on diet are difficult to distinguish from the effects of rainfall, because DFTD has spread from east to west in Tasmania, matching the rainfall gradient. Preliminary analysis revealed that rainfall and the presence of DFTD (0 = if absent at a site and 1 = if present at a site) were correlated (Pearson’s r–value = -0.75, p < 0.05, n = 12). As rainfall varied more continuously across Tasmania and our sites were either not affected by disease or diseased for more than seven years, we chose to include rainfall as the descriptor variable in analyses. For both devils and quolls, we performed a generalised linear mixed model (GLMM) for each prey category (using the ‘lme4’ library in R version 3.1.3 [49]). The average rainfall (mm) over the five years preceding collection of scats was included as a fixed factor and site was included as a random factor. We chose five years to allow for responses in prey population size to changes in rainfall. Rainfall was centred to avoid large correlation with sites.

## Results

We collected 660 Tasmanian devil scats from 10 sites and 177 spotted-tailed quoll scats from 7 sites (Table 1). Scats from both devils and quolls were collected at 4 sites (Meander, Freycinet, Arthur River and Cradle Mountain). Estimated dietary diversity for devils and quolls reached an asymptote with increasing sample size and showed that our sample sizes were more than adequate to represent dietary diversity for both species (S1 Fig).

### Diet composition

**Fig 2:**
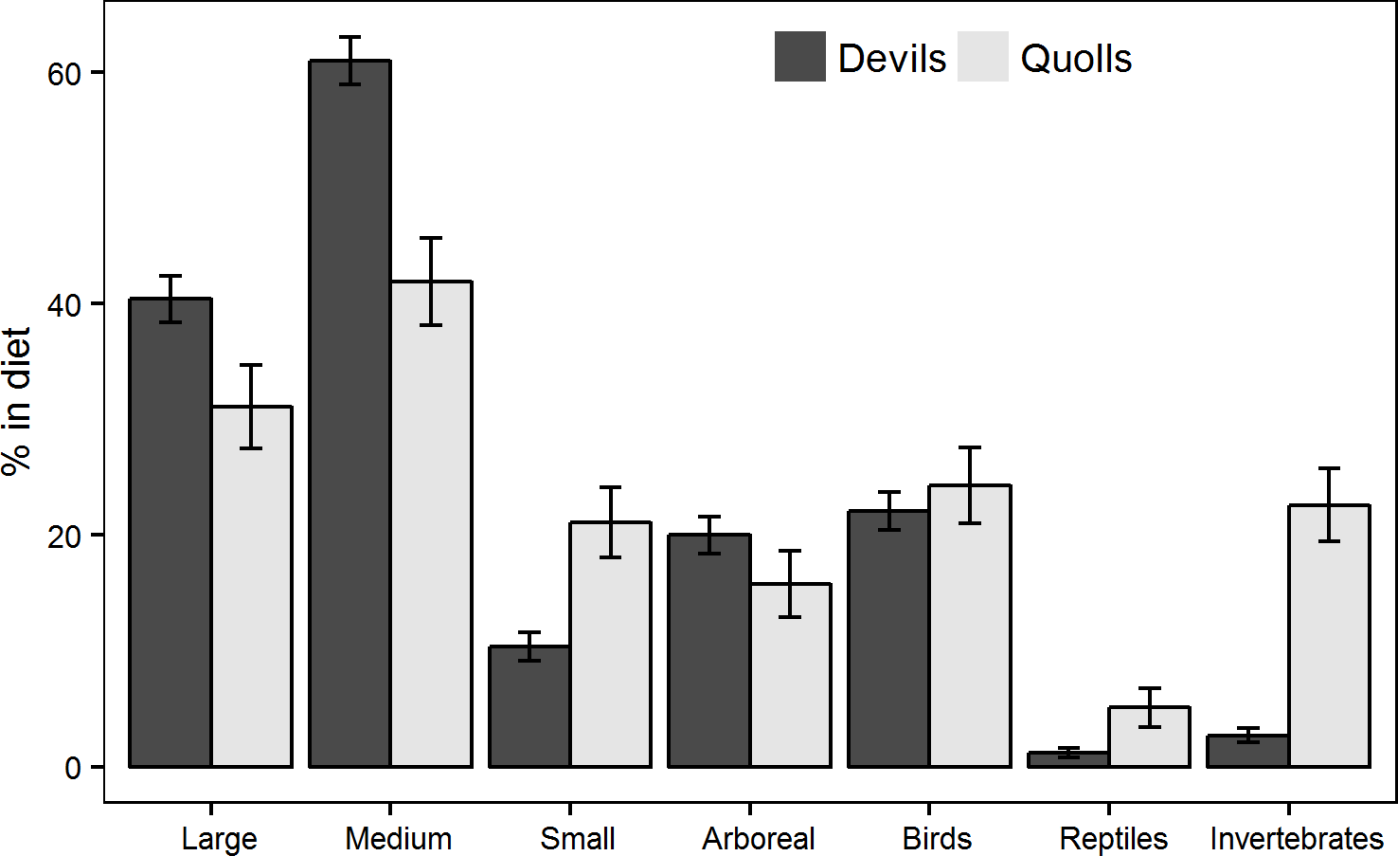
Frequency of occurrence (mean ± s.e.) of large, medium, small and arboreal mammalian prey species and birds, reptiles and invertebrates in devil and quoll scats.

Devils consumed a total of 26 prey taxa and quolls consumed a total of 22 prey taxa. All six major food categories were represented (Table 2). Mammals dominated the diet of both devils and quolls in terms of both frequency of occurrence and volume (Table 2), with 23 and 19 mammal species identified in the diet of devils and quolls, respectively (Table 2). Tasmanian pademelon and Bennett’s wallaby were the most important mammalian prey species in terms of frequency of occurrence and volume in the diet of both devils and quolls (Table 2). The most important prey group for both carnivores was medium-sized mammals, followed by large mammals and birds (Table 2). However, the frequency of occurrence of prey groups in devil and quoll scats differed. Devils consumed more large (χ^2^ = 2.72, *p* = 0.031) and medium mammals (χ^2^ = 8.17, *p* = 0.004) than did quolls (Table 2 and Fig 2). Conversely, quolls consumed significantly more small mammals (χ^2^ = 10.22, *p* = 0.001), reptiles (χ^2^ = 9.55, *p* = 0.002) and invertebrates (χ^2^ = 68.82, *p* = 0.041) than devils (Table 2 and Fig 2). Small mammals and birds occurred at intermediate frequencies in the diet of both devils and quolls, but in terms of volume constituted little in the bulk of scats (Table 2). Reptiles occurred at low frequencies in the diet of both devils and quolls (1.2% and 5.1%, respectively; Table 2). Invertebrates were recorded in extremely low frequencies in the diet of devils (2.7%) but at intermediate frequencies in the diet of quolls (22%) (Table 2). The frequency of occurrence of arboreal mammalian prey species was 15.8% in quoll scats and 20% in devil scats (Fig 2) but this difference was not significant (χ^2^ = 1.48, *p* = 0.221). Four devil scats from Cradle Mountain contained spotted-tailed quoll fur.

**Table 2:** Percent frequency occurrence (%F) and relative volume (%V) of prey items in the diets of Tasmanian devils (n = 902 prey items and 660 scats) and spotted-tailed quolls (n = 258 prey items and 177 scats), across Tasmania, Australia.

Common name	Scientific name	Devils		Quolls	
		%F	%V	%F	%V
**Large mammals**		**39.4**	**34.4**	**31.1**	**27.4**
Common wombat	*Vombatus ursinus*	12.6	10.6	1.1	1.1
Bennett’s wallaby	*Macropus rufogriseus*	24.5	22.4	27.7	24.3
Sheep	*Ovis aries*	0.6	0.3	1.7	1.5
Goat	*Capra hircus*	0.2	0.2	-	-
Cow	*Bos taurus*	0.6	0.4	0.6	0.5
Horse	*Equus caballus*	0.3	0.2	-	-
Dog	*Canis familiaris*	0.6	0.2	-	-
**Medium mammals**		**61.0**	**53.4**	**41.9**	**37.5**
Tasmanian pademelon	*Thylogale billardierii*	40.6	39.0	20.9	18.7
Brushtail possum	*Trichosurus vulpecula*	6.2	5.0	6.2	4.8
Ringtail possum	*Pseudocheirus peregrinus*	11.5	7.2	6.2	5.4
Bettong	*Bettongia gaimardi*	0.3	0.3	-	-
Potoroo	*Potorous tridactylus*	0.9	0.9	-	-
Southern brown bandicoot	*Isoodon obesulus*	0.5	0.3	1.1	1.4
Echidna	*Tachyglossus aculeatus*	0.8	0.4	1.1	1.1
Platypus	*Ornitohorhynchus anatinus*	-	-	0.7	0.6
Rabbit	*Oryctolagus cuniculus*	0.2	0.2	5.7	5.5
**Small mammals**		**10.4**	**5.05**	**21.1**	**16.3**
Water rat	*Hydromys chrysogaster*	0.3	0.3	-	-
Black rat	*Rattus rattus*	0.2	0.1	0.6	0.5
Swamp rat	*Rattus lutreolus*	0.3	0.1	1.7	1.7
House mouse	*Mus musculus*	-	-	0.6	0.6
Pygmy possum	*Cercartetus concinnus*	0.3	0.03	2.8	1.4
Sugar glider	*Petaurus breviceps*	2.0	0.9	0.6	1.1
Antechinus	*Antechinus* sp.	5.0	2.4	5.7	4.3
Long-tailed mouse	*Pseudomys higginsi*	2.1	1.2	5.7	5.2
White-footed dunnart	*Sminthopsis leucopus*	0.2	0.02	3.4	1.5
**Birds**		**22.1**	**7.0**	**24.3**	**11.0**
**Reptiles**		**1.2**	**0.1**	**5.1**	**1.0**
**Invertebrates**		**2.7**	**0.1**	**22.6**	**5.5**

### Trophic niche breadth and diet overlap

Niche breadth (B_A_) was greater for quolls than devils when data from all sites were pooled, and in all sites except for Freycinet (Table 3). Devil and quoll niche breadth was greatest at Cradle Mountain (Table 3). Niche breadth was lowest for devils at kunanyi/Wellington Park and lowest for quolls at Epping Forest (Table 3). The diet of devils and quolls overlapped extensively when data from all sites were pooled, and in all sites (Table 3).

**Table 3:** Trophic niche breadth (Levins’ index) and diet overlap (Pianka’s index) between Tasmanian devils and spotted-tailed quolls for each site in Tasmania, Australia.

Site	Devil	Quoll	Overlap
All	0.437	0.795	0.917
Arthur River	0.289	0.573	0.850
Freycinet	0.417	0.393	0.954
Cradle Mountain	0.542	0.848	0.779
Meander	0.335	0.483	0.866
wukalina/Mount William	0.195	-	-
Oldina	0.127	-	-
Ross	-	0.581	-
Epping Forest	-	0.326	-
Elderslie	0.271	-	-
Snug Tiers	0.194	-	-
Woolnorth	0.316	-	-
kunanyi/Wellington Park	0.033	-	-
Melaleuca	-	0.349	-

Female devils had a broader niche breadth than male devils at Freycinet, Cradle Mountain and Elderslie, whereas males had a broader niche breadth at Arthur River, Snug Tiers and Woolnorth (Table 4). Niche breadth was the same for both sexes at Oldina (Table 4). Male quolls had a broader niche than female quolls at Arthur River and Cradle Mountain, whereas females had a broader niche at Freycinet (Table 4). There was extensive diet overlap among all sex and species combinations at all sites except for a lower diet overlap between female quolls and devils of either sex at Cradle Mountain (Table 4).

**Table 4:** Trophic niche breadth (Levins’ index) for each sex (F = Females and M = Males) of Tasmanian devils (TD) and spotted-tailed quolls (STQ) and diet overlap (Pianka’s index) for each combination of sexes and species. Number of scats (n) for each sex at each site is included in parentheses.

		Arthur River	Freycinet	Cradle Mountain	Oldina	Elderslie	Snug Tiers	Woolnorth
TD	F	0.185 (70)	0.435 (16)	0.563 (163)	0.127 (14)	0.467 (7)	0.186 (19)	0.294 (23)
	M	0.281 (55)	0.340 (14)	0.520 (186)	0.127 (14)	0.103 (9)	0.210 (8)	0.310 (13)
STQ	F	0.448 (7)	0.400 (5)	0.501 (17)	-	-	-	-
	M	0.580 (30)	0.316 (6)	0.885 (58)	-	-	-	-
Overlap	TD_FM_	0.973	0.940	0.990	1.000	0.832	0.995	0.919
	STQ_FM_	0.916	0.861	0.842	-	-	-	-
	TD_M_-STQ_M_	0.835	0.998	0.836	-	-	-	-
	TD_F_-STQ_F_	0.928	0.857	0.487	-	-	-	-
	TD_M_-STQ_F_	0.930	0.865	0.488	-		-	-
	TD_F_-STQ_M_	0.768	0.917	0.838	-	-	-	-

### Geographic variation in diets

Diets of devils and quolls varied among sites but Tasmanian pademelon and Bennett’s wallaby were consistently important (S1 and S2 Tables). Rainfall had a significant effect on the diet composition of quolls but no effect on devils (Table 5). The occurrence of large mammals in the diet of quolls was higher, while small mammals and invertebrates were lower, with lower rainfall (Table 5), representing a decline both from east to west and with rising altitude.

**Table 5:** Generalised linear mixed model (GLMM) estimates and standard errors for the effect of rainfall on diet composition of devils and quolls for each prey category. Site was included as a random factor. Bold numbers indicate statistical significant (*p* value <0.05).

		Intercept	Rainfall			Site	
		Estimate ± s.e.	Estimate ± s.e.	z-value	p-value	Variance	Std
Devils	Large mammals	-1.38 ± 0.60	-0.14 ± 0.57	-0.25	0.806	1.10	1.05
	Medium mammals	1.18 ± 0.58	-0.01 ± 0.55	-0.01	0.993	1.14	1.07
	Small mammals	-3.66 ± 1.26	1.70 ± 1.04	1.62	0.105	3.34	1.83
	Birds	-1.26 ± 0.40	0.01 ± 0.40	0.01	0.984	0.57	0.75
	Invertebrates	-4.26 ± 0.37	0.65 ± 0.42	1.57	0.117	0	0
	Reptiles	-4.94 ± 0.57	1.05 ± 0.66	1.60	0.110	0	0
Quolls	Large mammals	-0.92 ± 0.18	-0.48 ± 0.18	-2.66	**0.008**	0	0
	Medium mammals	-0.37 ± 0.16	-0.18 ± 0.16	-1.09	0.278	0.09	0.29
	Small mammals	-1.42 ± 0.22	1.24 ± 0.26	4.77	**<0.001**	0.34	0.59
	Birds	-1.18 ± 0.19	-0.29 ± 0.19	-1.54	0.125	0.01	0.12
	Invertebrates	-1.31 ± 0.20	0.79 ± 0.22	3.61	**<0.001**	0	0
	Reptiles	-5.99 ± 15.79	9.30 ± 28.51	0.38	0.701	3.03	1.74

## Discussion

Devils and quolls show high overlap in dietary niche but with significant partitioning in prey size and a broader niche in quolls than in devils. Both species consume predominately macropods (Tasmanian pademelon and Bennett’s wallaby) and birds but also a wide range of prey species at lower frequencies, confirming that both species are opportunistic and flexible foragers [28, 30, 50, 51]. Devils consume larger prey than quolls. Rainfall, which is confounded with population decline of devils from facial tumour disease, influences the diet of quolls but not devils. In drier sites, which is also where devils have experienced the greatest population decline, quolls consume more large mammals than in wetter sites where devil density was still intact at the time the scats were collected. Extensive dietary overlap suggests high potential for both exploitation and interference competition over food resources between devils and quolls if resources become limited and for competitive release of quolls if devils are lost from the landscape.

Our study reveals resource partitioning based on prey size, despite substantial overlap in prey species in the diet, which could be explained by the difference in body size of the carnivores. Devils are larger and consume more large (e.g. wombats) and medium- sized mammals (e.g. pademelons), whereas the smaller quolls have a broader dietary niche and consume more small mammals, reptiles and invertebrates. Prey size partitioning amongst carnivores with different body sizes has been documented in other systems e.g. dingos/wild dogs (*Canis dingo/familiaris*) and coyotes (*Canis latrans*) consume more large mammals than red foxes (*Vulpes vulpes*) and swift foxes (*Vulpes velox*), respectively [9, 52]. Niche partitioning in prey size could also be a result of past competition (‘ghost of competition past’, e.g. Connell [53]) driving rapid evolution in trophic morphology [26, 54]. Competitive character displacement has minimised diet overlap within the constraints of the body size range of the two species (devils: 5–14 kg; quolls 0.9–5 kg), through evolution of equal spacing in canine tooth strength and jaw-closing musculature among sexes and species of quolls [54]. This has determined the upper size of prey that can be killed. Thus, we expect that this competitive character displacement has been maintained by ongoing episodic competition between devils and quolls, but which could be relaxed resulting in competitive release and rapid evolution in trophic structures if population abundance of the larger species, the devil, is reduced over a number of generations. We could also expect competitive release in prey-size selection of quolls on a shorter time-scale following disease-induced population decline of devils, allowing quolls to expand their niche to consume larger prey. This is consistent with our results and is a parsimonious explanation for the greater component of large-sized mammals and reduced component of small mammals and invertebrates in the diet of quolls in the drier eastern part of Tasmania, even though the east to west pattern of devil population decline is correlated with the rainfall gradient (increasing east to west). We propose that the greater proportion of larger prey in quoll diet represents increased scavenging of carcasses rather than an increase in killing of larger prey by quolls. Carcasses are a focus for competition between devils and quolls, and in such contests the larger devil is dominant and displaces the spotted-tailed quoll [55]. This result may indicate a greater availability of carrion in the landscape following the population decline of devils, the primary scavenger in Tasmanian ecosystems.

Knowing the context of food consumed in carnivore diet is important to establish whether dietary overlap could represent competition. The high diet overlap between devils and quolls may reflect either a partially commensal relationship, or consumption of macropod carrion through scavenging, or a difference in age class of macropod prey killed, or a combination of all three, but we are unable to distinguish these potential explanations in our study. First, larger predators can facilitate scavenging opportunities for smaller predators, as is suggested for brown hyenas (*Parahyaena brunnea*) scavenging on large herbivores killed by larger predators such as lions (*Panthera leo*) and wild dogs (*Lycaon pictus*) [56]. This commensal relationship may well describe part of the devil–quoll interaction. While quolls have been recorded killing adult male pademelons (~6-7kg) [57] it is not known how frequently they do this, but it is plausible that quolls scavenge on kills of macropods made by devils. Second, scavenging would lead to high diet overlap and both devils and quolls scavenge, although only devils have morphological specialisations for eating the tough parts of carcasses such as bones and thick skin [26]. The likely high availability of road-killed macropods at sites with high-speed sealed roads (Arthur River, Freycinet and Meander) may obscure differences in the size of prey consumed by these two carnivores. Similarly, the presence of large species of livestock (cows and sheep) in quoll scats probably represents consumption of carrion. Consumption of livestock carrion has been reported for other medium-sized carnivores, such as coyotes [58] and golden jackals (*Canis aureus*) [59]. Third, it is possible that quolls focus on killing small individuals and juveniles of larger prey species; juvenile macropods would be particularly vulnerable to predation by quolls from the time they begin to vacate the pouch until beyond independence. Vulnerable newborn animals are an important seasonal food source for other carnivores, such as red foxes [60] and wolverines (*Gulo gulo*) [61]. While the age of prey individuals consumed can often be recorded for devils by matching bone fragments in their scats with museum specimens [27], quolls do not consume large bones and so we could not determine the age of the macropod prey items in the diet.

Coexistence between Tasmanian devils and spotted-tailed quolls may be facilitated by the widespread distribution and high abundance of their dominant macropod prey species, Tasmanian pademelons and Bennett’s wallabies [62]. These macropods reach their highest abundance in fragmented agricultural landscapes [63, 64], which occur at or within 5km of all of our sites except Cradle Mountain, Melaleuca and kunanyi/Mt Wellington. Devils and quolls utilise edges in fragmented habitats to hunt macropods, which are vulnerable to predation as they cross the ecotone from native vegetation to pasture at dusk and return to native vegetation at dawn [65]. Carcasses of Bennett’s wallabies and pademelons also provide abundant carrion for devils and quolls, from animals that die of natural causes, from roadkill [65, 66] and from culling operations on farms to reduce competition with domestic livestock and damage to crops.

Vertical partitioning of resources can also enable sympatric carnivores to coexist [67, 68]. For example, the three of eight sympatric carnivores in central Africa with the highest dietary overlap showed temporal and vertical niche partitioning [13]. The long-nosed mongoose (*Herpestes naso*) is diurnal and ground-dwelling, the black-footed mongoose (*Bdeogale nigripes*) nocturnal and ground-dwelling while the servaline genet (*Genetta servalina*) is nocturnal and largely arboreal [13]. Spotted-tailed quolls have adaptations for tree-climbing, such as a clawless hallux on the pes and ridges on the foot pads, which are lacking in devils that are far less adept at climbing trees [26]. Despite this, we found no evidence of vertical niche partitioning of the diets of the two species. Quolls on the mainland of Australia, which have a high dietary overlap with sympatric red foxes and wild dogs, consume more arboreal prey, which could facilitate co-existence with those larger carnivores [69]. An explanation for the lack of partitioning in arboreal prey between quolls and devils in our study could be that there are few arboreal mammal species in Tasmania. Most species of arboreal mammals that occur on the adjacent mainland in Victoria did not occupy Tasmania or cross the arid Bassian land bridge during the Pleistocene [70]. Of the two arboreal mammals that are common in Tasmania, brushtail possums forage extensively on the ground and ringtail possums also frequent the ground, where they are available as prey to devils. Devils and quolls use the same vegetation types [65] but the ability of quolls to utilise the arboreal niche could give them a competitive advantage over devils should resources become limited.

Carnivore species that have high dietary overlap are more likely to encounter each other as they seek similar prey [16]. These interactions can result in interference competition over the contested resource, including aggravated aggression leading to intraguild killing. Differences in body size can influence the outcome of these interactions [16]. The body-size difference between devils and quolls is in the intermediate range (e.g., the larger predator is 2–5 times bigger than the smaller species), within which the likelihood of intense interspecific aggression, including killing, is expected to be high [16]. Nonetheless, we found only four cases of significant amounts of quoll fur in devil scats and no devil fur in quoll scats. We are not able to determine if the quoll fur represented intraguild predation or was a result of devils scavenging on quolls. The extent of intraguild killing between devils and quolls is poorly known but anecdotal observations provide evidence that devils do kill quolls and quolls can inflict injury on devils in contests over carcasses [71]. Quolls become vigilant when they encounter a devil scat, which suggests that they might fear and avoid interspecific aggression [72]. The frequency of intraguild killing may be underestimated in diet studies, as animals killed in acts of extreme aggressive interference competition are not always consumed [73]. For example, coyotes do not always consume San Joaquin kit foxes (*Vulpes macrotis*) that they killed [74]. This suggests that minimizing competition is more important for coyotes than obtaining any nutritional benefits from kit foxes.

While we found evidence consistent with competitive release of quolls in areas that have been subject to devil decline, even if this represents an expansion of the scavenging of larger prey niche, this does not appear to have resulted in an increase in population size [75]. A space-for-time study using hair traps indicated that quolls are at much lower densities in regions where there has been long-term devil decline relative to adjacent regions with more recent decline [34]. Continuation of these studies that include a temporal component will reveal just how quoll populations are responding to devil decline.

Population decline of devils could also result in the competitive release of feral cats, and there is increasing evidence that feral cats are now increasing in activity and population size [33, 34]. The similar body size and prey composition of feral cats and spotted-tailed quolls suggest that interspecific competition is likely to occur [27, 55, 76], and there are reports of both species killing one another [77]. As feral cats are able to breed twice per year, compared to once for quolls, cats may be able to outcompete quolls demographically and so competitive release of cats may counter any competitive release that might benefit quolls [34]. Furthermore, if intraguild competition is weak, or if bottom-up forces are strong as could be expected in productive environments [78] such as the agricultural regions of Tasmania [34], the decline of the top predator will have only negligible effects on mesopredators and may not result in an increased abundance.

Co-existence between the two hypercarnivorous members of the Australian endemic carnivorous marsupial fauna, the Tasmanian devil and the spotted-tailed quoll, appears to be facilitated by the widespread distribution and availability of medium-sized macropod species, as both live prey and as carrion, and the ability of the smaller quoll to utilise a broad range of prey species. A high dietary overlap suggests that exploitation and interference competition could occur should resources become limited [18]. Conversely, the severe and sustained population decline of the Tasmanian devil from disease may be triggering competitive release of quolls, at least in the scavenging niche, although a complex series of trophic interactions involving the similar-sized invasive feral cat may counter any benefit for quolls.

## Supporting information

S1 Fig Brillouin diversity index of devil (TD) and quoll (STQ) diets with increasing sample size of scats across Tasmania, Australia. (TIFF)

S1 Table Percent frequency occurrence (%F) and relative volume (%V) of prey items, in Tasmanian devil scats for each site. (PDF)

S2 Table Percent frequency occurrence (%F) and relative volume (%V) of prey items, in spotted-tailed quoll scats for each site. (PDF)
