# Supplementary figures and images for: Dietary partitioning of Australia's two marsupial hypercarnivores, the Tasmanian devil and the spotted-tailed quoll, across their shared distributional range
Source: PLoS One. 2017 Nov 27;12(11):e0188529. doi: 10.1371/journal.pone.0188529 (PMC5703475; doi:10.1371/journal.pone.0188529)

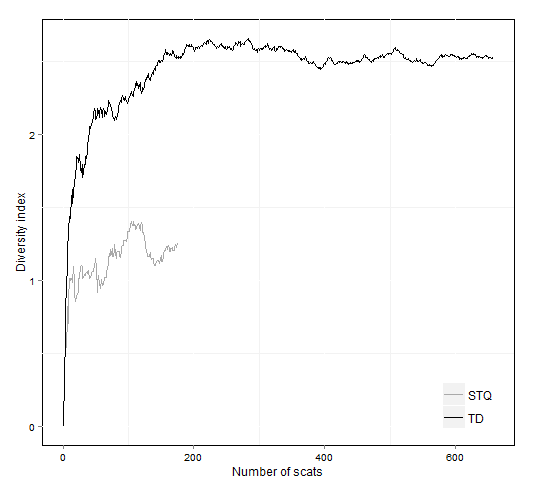

Supplement: S1 Fig — (TIFF) [file pone.0188529.s001.tiff]
