# Supplementary material for: Dietary partitioning of Australia's two marsupial hypercarnivores, the Tasmanian devil and the spotted-tailed quoll, across their shared distributional range
Source: PLoS One. 2017 Nov 27;12(11):e0188529. doi: 10.1371/journal.pone.0188529 (PMC5703475; doi:10.1371/journal.pone.0188529)
Supplement: S1 Table — (PDF) [file pone.0188529.s002.pdf]

Common name	Scientific name	Arthur River		Woolnorth		Freycinet		Elderslie		Wellington park		Mount William		Meander		Oldina		Snug Tiers		Cradle Mountain	
		%F	%V	%F	%V	%F	%V	%F	%V	%F	%V	%F	%V	%F	%V	%F	%V	%F	%V	%F	%V
**Large Mammals**		32.8	32.5	33.3	32.5	60.0	45.1	18.8	18.1	7.7	7.7	14.3	12.8	38.1	38.8	0	0	7.4	6.7	49.1	40.6
Common wombat	*Vombatus ursinus*	2.4	2.4	0	0	0	0	0	0	0	0	0	0	0	0	0	0	0	0	22.9	19.1
Bennett's wallaby	*Macropus rufogriseus*	30.4	30.1	33.3	32.5	50.0	42.7	18.8	18.1	7.7	7.7	14.3	12.8	27.6	27.3	0	0	7.4	6.7	23.5	20.4
Sheep	*Ovis aries*	0	0	0	0	10.0	2.4	0	0	0	0	0	0	3.5	3.5	0	0	0	0	0	0
Goat	*Capra hircus*	0	0	0	0	0	0	0	0	0	0	0	0	3.5	3.5	0	0	0	0	0	0
Cow	*Bos taurus*	0	0	0	0	0	0	0	0	0	0	0	0	3.5	3.5	0	0	0	0	0.9	0.4
Horse	*Equus caballus*	0	0	0	0	0	0	0	0	0	0	0	0	0	0	0	0	0	0	0.6	0.3
Dog	*Canis familiaris*	0	0	0	0	0	0	0	0	0	0	0	0	0	0	0	0	0	0	1.2	0.4
**Medium Mammals**		67.2	66.2	66.7	64.8	56.7	45.1	75.0	73.8	91.9	91.9	85.7	84.3	51.9	51.8	100.1	96.5	92.6	88.9	51.3	38.9
Tasmanian pademelon	*Thylogale billardierii*	61.6	60.7	61.1	59.2	36.7	32.9	75.0	73.8	76.9	76.9	85.7	84.3	27.6	27.6	92.9	89.3	92.6	88.9	20.3	18.6
Brushtail possum	*Trichosurus vulpecula*	0.8	0.7	5.6	5.6	10.0	7.9	0	0	15.4	15.4	0	0	10.4	10.3	3.6	3.6	0	0	8.3	6.3
Ringtail possum	*Pseudocheirus peregrinus*	0	0	0	0	0	0	0	0	0	0	0	0	0	0	3.6	3.6	0	0	21.5	13.3
Bettong	*Bettongia gaimardi*	0	0	0	0	0	0	0	0	0	0	0	0	6.9	6.9	0	0	0	0	0	0
Potoroo	*Potorous tridactylus*	3.2	3.2	0	0	3.3	3.4	0	0	0	0	0	0	3.5	3.5	0	0	0	0	0	0
Southern brown	*Isoodon*	0	0	0	0	0	0	0	0	0	0	0	0	3.5	3.5	0	0	0	0	0.6	0.3

bandicoot	*obesulus*																				
Echidna	*Tachyglossus aculeatus*	0.8	0.8	0	0	6.7	0.9	0	0	0	0	0	0	0	0	0	0	0	0	0.6	0.4
Rabbit	*Oryctolagus cuniculus*	0.8	0.8	0	0	0	0	0	0	0	0	0	0	0	0	0	0	0	0	0	0
**Small mammals**		0	0	0	0	0	0	6.3	5.6	0	0	0	0	10.4	10.1	0	0	0	0	18.4	8.7
Water rat	*Hydromys chrysogaster*	0	0	0	0	0	0	0	0	0	0	0	0	6.9	6.6	0	0	0	0	0	0
Black rat	*Rattus rattus*	0	0	0	0	0	0	6.3	5.6	0	0	0	0	0	0	0	0	0	0	0	0
Swamp rat	*Rattus lutreolus*	0	0	0	0	0	0	0	0	0	0	0	0	0	0	0	0	0	0	0.6	0.2
Antechinus	*Antechinus* sp.	0	0	0	0	0	0	0	0	0	0	0	0	0	0	0	0	0	0	9.5	4.5
Long-tailed mouse	*Pseudomys higginsi*	0	0	0	0	0	0	0	0	0	0	0	0	0	0	0	0	0	0	4.0	2.3
White-footed dunnart	*Sminthopsis leucopus*	0	0	0	0	0	0	0	0	0	0	0	0	0	0	0	0	0	0	0.3	0.1
Pygmy possum	*Cercartetus concinnus*	0	0	0	0	0	0	0	0	0	0	0	0	0	0	0	0	0	0	0.6	0.1
Sugar glider	*Petaurus breviceps*	0	0	0	0	0	0	0	0	0	0	0	0	3.5	3.5	0	0	0	0	3.4	1.5
**Birds**		8.8	1.1	27.8	2.8	46.7	9.3	25.0	2.5	0	0	28.6	2.9	6.9	0.7	35.7	3.6	44.4	4.5	23.2	10.9
**Reptiles**		0.8	0.2	0	0	0	0	0	0	0	0	0	0	0	0	0	0	0	0	2.0	0.2
**Invertebrates**		0	0	0	0	3.3	0.3	0	0	0	0	0	0	0	0	0	0	0	0	4.6	0.7
No. items		138		46		48		20		13		9		31		38		39		518	
No. scats		125		36		30		16		13		7		29		28		27		349	
