# Supplementary material for: Dietary partitioning of Australia's two marsupial hypercarnivores, the Tasmanian devil and the spotted-tailed quoll, across their shared distributional range
Source: PLoS One. 2017 Nov 27;12(11):e0188529. doi: 10.1371/journal.pone.0188529 (PMC5703475; doi:10.1371/journal.pone.0188529)
Supplement: S2 Table — (PDF) [file pone.0188529.s003.pdf]

Common name	Scientific name	Arthur River		Epping forest		Freycinet		Melaleuca		Ross		Meander		Cradle Mountain	
		%F	%V	%F	%V	%F	%V	%F	%V	%F	%V	%F	%V	%F	%V
**Large mammals**		38.9	33.3	35.3	34.7	54.6	45.5	0	0	12.5	6.3	63.2	55.3	21.3	17.6
Common wombat	*Vombatus ursinus*	0	0	0	0	9.1	9.1	0	0	0	0	0	0	1.3	1.2
Bennett's wallaby	*Macropus rufogriseus*	38.9	33.3	23.5	22.9	36.4	36.4	0	0	12.5	6.3	57.9	51.6	20.0	16.4
Sheep	*Ovis aries*	0	0	11.8	11.8	0	0	0	0	0	0	5.3	3.7	0	0
Cow	*Bos taurus*	0	0	0	0	9.1	7.3	0	0	0	0	0	0	0	0
**Medium mammals**		44.5	59	58.8	57.8	36.4	31.8	80.0	58.9	50.0	50.0	31.6	31.4	34.6	30.2
Tasmanian pademelon	*Thylogale billardierii*	36.1	30.0	0	0	18.2	18.2	70.0	48.9	37.5	37.5	26.3	26.2	9.3	9.3
Brushtail possum	*Trichosurus vulpecula*	0	0	5.9	5.8	18.2	13.6	0	0	12.5	12.5	0	0	9.3	6.7
Ringtail possum	*Pseudocheirus peregrinus*	0	21.2	0	0	0	0	0	0	0	0	0	0	14.7	12.9
Southern brown bandicoot	*Isoodon obesulus*	5.6	5.4	0	0	0	0	0	0	0	0	0	0	0	0
Echidna	*Tachyglossus aculeatus*	0	0	0	0	0	0	10.0	10.0	0	0	0	0	1.3	1.3
Platypus	*Ornitohorhynchus anatinus*	0	0	0	0	0	0	0	0	0	0	5.3	5.2	0	0
Rabbit	*Oryctolagus cuniculus*	2.8	2.4	52.9	51.8	0	0	0	0	0	0	0	0	0	0
**Small mammals**		8.4	5.7	0	0	0	0	10.0	10.0	25.0	15.0	5.3	5.0	39.9	34.4
Black rat	*Rattus rattus*	0	0	0	0	0	0	0	0	0	0	5.3	5.0	0	0
Swamp rat	*Rattus lutreolus*	5.6	5.4	0	0	0	0	10.0	10.0	0	0	0	0	0	0

House mouse	*Mus musculus*	0	0	0	0	0	0	0	0	12.5	12.5	0	0	0	0
Antechinus	*Antechinus* sp.	0	0	0	0	0	0	0	0	0	0	0	0	13.3	10.4
Long-tailed mouse	*Pseudomys higginsi*	0	0	0	0	0	0	0	0	0	0	0	0	13.3	12.5
White-footed dunnart	*Sminthopsis leucopus*	0	0	0	0	0	0	0	0	0	0	0	0	8.0	8.2
Pygmy possum	*Cercartetus concinnus*	0	0	0	0	0	0	0	0	12.5	2.5	0	0	5.3	3.3
Sugar glider	*Petaurus breviceps*	2.8	0.3	0	0	0	0	0	0	0	0	0	0	0	0
**Birds**		41.2	21.2	23.5	7.7	18.2	13.6	20.0	0.5	37.5	17.5	21.1	5.6	17.3	8.4
**Reptiles**		0	0	0	0	0	0	0	0	0	0	0	0	12.0	2.4
**Invertebrates**		11.1	1.9	0	0	9.1	1.8	70.0	20.6	12.5	11.3	15.8	2.7	32.0	7.1
No. items		52		20		13		18		11		26		118	
No. scats		36		17		11		10		8		19		75	
